# Efficacy and safety of a fixed‐dose combination of ibuprofen and caffeine in the management of moderate to severe dental pain after third molar extraction

**DOI:** 10.1002/ejp.1068

**Published:** 2017-08-14

**Authors:** T. Weiser, E. Richter, A. Hegewisch, D.D. Muse, R. Lange

**Affiliations:** ^1^ Medical Affairs Consumer Health Care, Medical and Regulatory Affairs Germany Boehringer Ingelheim Pharma GmbH & Co. KG Germany; ^2^ Corporate Division Medicine Global Department Biostatistics and Data Sciences Boehringer Ingelheim Pharma GmbH & Co. KG Germany; ^3^ Marketing Self‐Medication Global Department Consumer Health Care Division Medical and Regulatory Affairs Boehringer Ingelheim, Promeco S.A. de C.V Germany; ^4^ Jean Brown Research Salt Lake City USA; ^5^ Consumer Health Care Development Medical and Regulatory Affairs Boehringer Ingelheim Pharma GmbH & Co. KG Germany

## Abstract

**Background:**

Ibuprofen is an effective analgesic treatment with a ceiling effect at doses above 400 mg. This study compared the combination of ibuprofen 400 mg and caffeine 100 mg with ibuprofen 400 mg monotherapy, caffeine and placebo in the analgesic treatment of moderate to severe acute dental pain following third molar extraction.

**Methods:**

Phase III, active‐/placebo‐controlled, double‐blind, single‐centre, two‐stage, parallel‐group study in adult patients with at least moderate baseline pain intensity. Primary endpoint was defined as the time‐weighted sum of pain relief and pain intensity difference over 8 h (SPRID0–8 h), secondary endpoints included duration of pain relief, time to meaningful pain relief and more.

**Results:**

*N* = 748 patients were enrolled and *N* = 562 treated. Mean baseline pain intensity was 7.7 on a 0–10 numerical rating scale. Analysis of SPRID0–8 h demonstrated superior analgesic effects for a single dose of ibuprofen/caffeine versus ibuprofen, caffeine and placebo over 8 h, rescue medication in this stage was requested by more patients on ibuprofen (32.5%) than on ibuprofen/caffeine (16.0%). Median time to meaningful pain relief was shorter for ibuprofen/caffeine (1.13 h) compared with ibuprofen (1.78 h; *p* = 0.0001). More patients on ibuprofen/caffeine than on ibuprofen reported meaningful pain relief. Adverse events were infrequent and mostly mild or moderate across treatment groups. Tolerability was rated as ‘very good’ or ‘excellent’ by most patients in both treatment groups.

**Conclusion:**

This study demonstrated clinically relevant superiority of ibuprofen/caffeine over monotherapy with ibuprofen in patients with acute dental pain. All treatments were well tolerated.

**Significance:**

This trial showed superior efficacy of 400/100 mg ibuprofen/caffeine, compared to 400 mg ibuprofen alone, for treating acute pain, reflecting that caffeine is an effective analgesic adjuvant. Data on efficacy of 400 mg ibuprofen combined with caffeine for the treatment of acute pain were not available yet.

## 1 Introduction

The non‐steroidal anti‐inflammatory drug (NSAID) ibuprofen was first approved in 1968 in the UK and its favourable efficacy and safety profile have been well established over almost half a century of clinical use. After oral administration of a single dose analgesia is achieved rapidly and maintained for up to 8 h. Its well‐proven efficacy and the beneficial safety profile – especially for the short‐term treatment of acute pain – make ibuprofen one of the most popular analgesics.

At single doses exceeding 400 mg, ibuprofen has pronounced anti‐inflammatory effects, which are used for, e.g. long‐term treatment of chronic inflammatory diseases (like rheumatism; Rainsford, 2009). However, clinical studies have shown that analgesic efficacy for the treatment of acute pain is not increased by the administration of higher doses than 400 mg. This ‘ceiling‐effect’ of ibuprofen has been observed in studies investigating postsurgical, as well as migraine pain (Laska et al., 1986; Seymour et al., 1996; Kellstein et al., 2000).

Since decades analgesic compounds have been combined with caffeine as adjuvans, and recent meta‐analyses have proven that the addition of 100–130 mg caffeine to a standard dose of an analgesic induce a clinically relevant increase in the number of patients who benefit from treatment (Derry et al., 2014, 2015). This adjuvant effect has been shown for single agents (like the NSAIDs aspirin or ibuprofen, or paracetamol), as well as for the combination of aspirin and paracetamol with caffeine. Caffeine is an antagonist at adenosine receptors, which are assumed to be relevant for pain signal processing und transmission (Sawynok, 2011).

Up to now it has not been clinically investigated whether the addition of 100 mg caffeine to the maximum effective dose of ibuprofen for treating an acute pain event (i.e. 400 mg) increases efficacy, compared to ibuprofen alone.

This double‐blind clinical study was conducted to assess the acute effect of the combination of ibuprofen (acid) 400 mg and caffeine 100 mg in patients with post‐surgical dental pain. The primary objective was to demonstrate superior efficacy of a single dose of ibuprofen/caffeine versus either ingredient alone as well as placebo. The dental impaction pain model is validated, reproducible and widely recognized and utilized as acute pain model for testing efficacy of analgesic treatments in clinical trials (Cooper and Desjardins, 2010; Singla et al., 2014).

## 2 Patients and methods

### 2.1 Design

This was a Phase III randomized, active‐ and placebo‐controlled, double‐blind, single‐centre, two‐stage, 6‐arm, parallel‐group study, comparing the effect of the fixed‐dose combination (FDC) of ibuprofen (acid) 400 mg and caffeine 100 mg versus ibuprofen (acid) 400 mg, caffeine 100 mg, and placebo in patients aged between 18 and 55 with postoperative dental pain. The study was conducted and reported in accordance with the Declaration of Helsinki, the ICH‐GCP guidelines, local regulations and Boehringer Ingelheim standard operating procedures (SOP)s, and in compliance with the clinical trial protocol. The study received prior ethics‐committee approval by the Institutional Review Board (IRB) of the participating centre (Chesapeake IRB, Columbia, MD, USA).

Electronic case report forms (eCRFs) were used for all patients. All clinical data were captured using the Oracle Clinical™ remote data capture (RDC) system, a web‐based tool. Data entered in the eCRFs had to be consistent with the source documents which were filed at the investigator's site. The investigator was responsible for retaining all records pertaining to the trial. Concomitant diagnoses and AEs were coded using the Medical Dictionary for Regulatory.

Activities (MedDRA) version 17.0 and concomitant medications were coded according to the World Health Organisation Drug Dictionary version 14.MAR.

A physical examination, ECG and laboratory tests were conducted at the screening visit to evaluate whether the patients were in good general health and thus suitable for study participation. Laboratory analyses were performed by Quest Diagnostics Clinical Trials, Valencia, California, USA. The investigator assessed the clinical significance of screening safety laboratory results to determine if the patient was healthy enough for study participation. Parameters included clinical chemistry, complete blood cell count, drug abuse test (urine dipstick), urinalysis and urine dipstick‐pregnancy test.

### 2.2 Ethical considerations

Before the start of the study, the clinical trial protocol (CTP), the patient information leaflet, the informed consent form and other locally required documents were reviewed by the Institutional Review Board (IRB) of the participating centre. The IRB (Chesapeake IRB, Columbia, MD, USA) of the principal investigator of the trial (Derek D. Muse, MD, Salt Lake City, Utah, USA) granted approval of the study on 02 Aug 2013. The IRB met the requirements of the International Conference on Harmonisation (ICH) Harmonised Tripartite Guideline for Good Clinical Practice (GCP), local legislation, and the requirements of 21 CFR 312.120. There was one global amendment to the CTP which required approval of the IRB.

### 2.3 Patients and treatment

This trial was performed in male or female outpatients who were eligible for participation in the study if they were between 18 and 55 years of age and scheduled to undergo surgical extraction of 3–4 impacted third molars, with a minimum of two mandibular extractions, were in good general health, with a body mass index (BMI) ≤30, had no contraindications to any of the study medications or anaesthetic drugs, and gave written informed consent before any pre‐screening, screening or study‐specific procedures were performed.

Patients qualified for the study if after the dental surgical procedure they presented a baseline pain severity assessment of either ‘moderate’ or ‘severe’ pain on a 4‐point verbal rating scale (VRS) which was based on a written selection of options of ‘no pain’, ‘slight pain’, ‘moderate pain’, or ‘severe pain’ and a baseline score of ≥5 on the 0–10 numerical pain rating scale (NPRS) ranging from 0 = ‘no pain’ to 10 = ‘worst possible pain’. Patients were periodically asked to verbally rate their pain using the NPRS, beginning about 30 min following completion of the surgical procedure, until the patient qualified with a NPRS pain intensity (PI) score of ≥5 or until 5 h had elapsed. The first dose of trial medication was administered no later than 5 min after the qualifying pain score assessment. Patients with ‘no pain’ or ‘slight pain’ on the VRS or with a score of <5 on the NPRS were not eligible for randomization. If the patient failed to qualify by 5 h post‐surgery, the patient was not randomized and considered a screening failure.

Patients were excluded from the study if they met any exclusion criteria, i.e. had a history of hypersensitivity to the study medications, any significant disease or gastrointestinal disorder, impaired liver function, clinically significant abnormal electrocardiogram (ECG) at screening, in case of alcohol or substance abuse, habituation to and abuse of other analgesic or psychotropic drugs, or ingested any caffeine‐containing beverages, chocolate, or alcohol 6 h or less before surgery. Women were excluded if pregnancy or breastfeeding could not be ruled out.

Trial medication (i.e. ibuprofen 400 mg/caffeine 100 mg, ibuprofen 400 mg, caffeine 100 mg, or placebo) was supplied as identically appearing film‐coated tablets. It was produced by Delpharm SAS, Reims, France and provided by the Clinical Trial Supplies Unit, BI Pharmaceuticals, Inc., Ridgefield, CT, USA. The batch release of trial kits was performed by the Department of Pharmaceutical R&D, Clinical Trial Supplies Unit, BI Pharma GmbH & Co. KG, Biberach, Germany.

The randomization list was generated using a validated system, which involved a pseudo‐random number generator so that the resulting treatment was both reproducible and non‐predictable. Patients were randomized in blocks to ensure that equal numbers of patients were allocated to each treatment sequence. A block size of 16 was used. Patients and investigators remained blinded with regard to the randomized treatment assignments until after database lock. Access to the codes was controlled and documented.

At the time of randomization, to stratify the randomization regarding the baseline pain intensity, eligible patients having reported ‘moderate’ were assigned the lowest available medication number, whereas patients having recorded ‘severe’ were assigned the highest available medication number. Interactive Voice Response (IVR) system was not utilized. After a screening evaluation (Visit 1), eligible patients underwent dental surgery at Visit 2 and were randomized to one of the six treatment sequences of the double‐blind treatment phase consisting of two study stages in a 6:6:1:1:1:1 ratio (Table 1).

**Table 1:** Randomization of patients to Study Stage 1 and 2

Study Stage 1	Study Stage 2
Ibuprofen/caffeine	Ibuprofen/caffeine
Ibuprofen	Ibuprofen
Caffeine	Ibuprofen/caffeine
Caffeine	Ibuprofen
Placebo	Ibuprofen/caffeine
Placebo	Ibuprofen

In Stage 1 of this study, patients received either one single dose of ibuprofen/caffeine, ibuprofen, caffeine or placebo and in Stage 2 patients received multiple doses of either ibuprofen/caffeine or ibuprofen according to the randomization scheme above.

**Figure 1:**
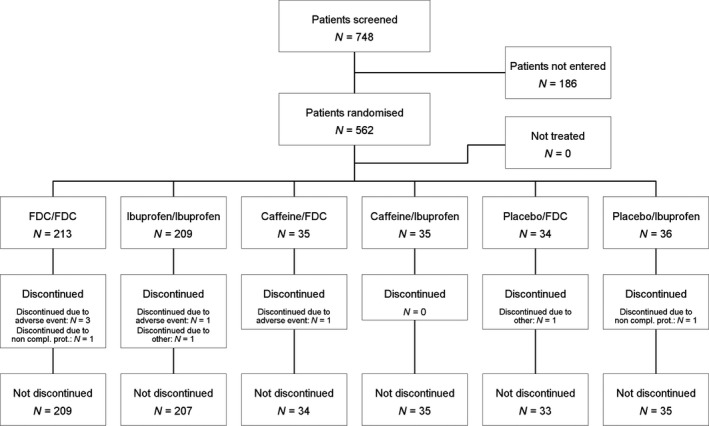
Disposition of patients by treatment sequence. Treatments for the two study stages are separated by slashes.

The disposition of patients is shown in Fig. 1.

Study Stage 1 was scheduled to last 8 h; however, if a patient requested rescue medication or the second dose of trial medication between 6 and 8 h after the first dose, Study Stage 1 ended at the time of the respective medication. After the first dose of trial medication, the patients remained at the trial site to be observed over an 8‐h time period. Patients were encouraged not to take rescue medication within the first 90 min after administration of trial medication. Protocol‐defined rescue medications were paracetamol 500–1000 mg (1–2 tablets) and paracetamol 500 mg plus hydrocodone 5 mg (1–2 tablets). After Study Stage 1, patients continued treatment with either ibuprofen/caffeine or ibuprofen on an outpatient basis for 5 days in Study Stage 2, with up to 3 doses per day every 6–8 h.

The study included three visits: Screening Visit (Visit 1), Dental Surgery/Study Stage 1 (Visit 2) and End‐of‐trial Visit (Visit 3). Participation in the trial started with the Screening Visit and ended with the End‐of‐trial Visit. Adverse events were to be recorded further during a follow‐up period, which comprised 7 days after the last administration of trial medication.

### 2.4 Endpoints

All assessments concerning the primary and secondary efficacy endpoints were completed in Study Stage 1 after single dosing of ibuprofen/caffeine, ibuprofen, caffeine or placebo over an 8‐h postoperative period on an inpatient basis. The primary endpoint of this trial was the outcome measure SPRID0–8 h: the time‐weighted sum of pain relief and pain intensity difference from pre‐dose baseline pain intensity – summed up for all assessment times from 0 to 8 h after administration of the first dose of study medication.

Pain relief from starting pain was assessed in a patient diary using a 5‐point VRS (0 = ‘none’, 1 = ‘a little’, 2 = ‘some’, 3 = ‘a lot’, 4 = ‘complete’) at 0.25, 0.5, 0.75, 1, 1.5, 2, 3, 4, 5, 6, 7 and 8 h after the first dose of study medication. Pain intensity was assessed using a 0–10 NPRS ranging from 0 = ‘no pain’ to 10 = ‘worst possible pain’. Patients assessed their dental pain intensity in a diary pre‐dose and at 0.25, 0.5, 0.75, 1, 1.5, 2, 3, 4, 5, 6, 7 and 8 h after the first dose of study medication. As soon as the patient required rescue medication or a second dose of study medication before 8 h post‐dose (whichever was first) pain intensity and pain relief were assessed before use of rescue/second intake of study medication. Subsequent pain intensity assessments after rescue/second intake of study medication were still performed.

Secondary endpoints comprised SPRID0–2 h (time‐weighted sum of pain relief and pain intensity difference from pre‐dose baseline pain intensity – summed up for all assessment times from 0 to 2 h after first dose of study medication), duration of pain relief and time to meaningful pain relief. Duration of pain relief was defined by the time to first dose of rescue medication or second dose of study medication (whichever was first) within the first 8 h after the first study drug intake.

Time to meaningful pain relief was captured by a stopwatch started immediately after the first dose of study medication and stopped as soon as a meaningful pain relief was felt by the patient.

Other endpoints included time to first perceptible pain relief, pain intensity difference (PID) and pain relief (PAR) at individual time points and number of rescue medication doses in Stage 1.

Time to first perceptible pain relief was captured by a stopwatch stopped as soon as the patient first began to feel any pain relief.

Safety was assessed based on the incidence and intensity of adverse events (AEs and SAEs) which were recorded from the time of consent until 7 days after completion of the study treatment course as well as on laboratory assessments, physical examinations and patients’ final global assessment of tolerability. This final global assessment of tolerability was performed by the patient at the end of the study by answering the question: ‘How would you rate your ability to tolerate the study medication’ (0 = ‘poor’, 1 = ‘fair’, 2 = ‘good’, 3 = ‘very good’, 4 = ‘excellent’) in the diary.

### 2.5 Statistical methods

For SPRID0–8 h, the treatment difference of the FDC versus the single components and placebo were tested using two‐sided tests and a significance level of 0.05. Secondary endpoints were considered as supportive and other endpoints as explorative endpoints, respectively.

The sample size calculation was based on SPRID0–8 h evaluated in Study Stage 1, in which the treatment groups FDC, ibuprofen, caffeine and placebo were allocated in a 3:3:1:1 ratio. A total of 560 evaluable patients (210:210:70:70) had 90% power to detect a standardized mean difference (SMD) of 0.45 when the sample sizes in the two groups were 70 and 210, respectively (a total sample size of 280), and 94% power to detect an effect size of 0.35, when the sample sizes in the two groups were 210 each. Therefore, a total planned number of 560 patients had to be randomized to one of the six treatment sequences as outlined in Fig. 1.

In total, three analysis datasets were defined: the treated set (TS), the full analysis set (FAS) and the per‐protocol set (PPS). The TS comprised all randomized patients who took at least one dose of study medication. The FAS included all patients in the TS who provided any post‐treatment data for the primary efficacy endpoint. The PPS was based on all patients in the FAS who had no important protocol violations.

SPRID0–8 h and SPRID0–2 h were tested for the FAS using an analysis of covariance (ANCOVA) including treatment as fixed effect and the pre‐dose baseline pain intensity measured on the 4‐point VRS as a categorical covariate. In these analyses, assessments of pain relief and pain intensity were considered as missing if completed after the patient had taken rescue medication or the second dose of study medication (whichever was first) before hour 8 after study drug intake. The last assessment completed before to rescue/second study medication was then carried forward to replace the observations up to 8 h.

The Kaplan–Meier estimator was presented for the endpoints ‘time to perceptible pain relief’, ‘time to meaningful pain relief’ and ‘duration of pain relief’ with the log rank test used to evaluate the difference between the treatment groups.

The analysis of PID at each time point utilized a restricted maximum likelihood‐based repeated measures approach, using all available longitudinal pain intensity observations or replaced‐by‐missing observations after the patient had taken rescue medication or the second dose of study medication (no LOCF procedure was applied in this repeated measures model) at each post‐baseline time up to 8 h. PID means were adjusted for the continuous covariate of baseline PI (NPRS).

An ordinal logistic regression model including the factors treatment, time and treatment‐by‐time interaction as well as the categorical covariate of baseline PI [VRS] was used for the analysis of PAR at each time point. In this analysis, all pain relief assessments completed during the first 8 h, after the patient had taken rescue or the second study medication, were set missing. Odds ratios together with 95% CIs were used to quantify the effect of the FDC compared to ibuprofen, caffeine and placebo; odds ratios > 1 were to be interpreted as being in favour of the FDC.

*Post hoc* responder analyses were performed with regard to SPRID0–8 h and the parameter SPRID0–6 h. A responder was defined as a patient who achieved 50% of the individual maximum achievable effect with regard to SPRID0–8 h/SPRID0–6 h, respectively. The maximum achievable SPRID0–8 h/SPRID0–6 h, respectively, was reached if PI was 0 on the 0–10 NPRS and PAR was 4 (=complete) at all assessment times under consideration.

Proportions of responders were used to calculate the absolute risk reduction (ARR) and the number needed to treat (NNT) for the FDC group in comparison to the ibuprofen, caffeine and placebo groups; in addition 95% confidence intervals (CI) by Wald were calculated for ARRs and NNTs.

All patients in the TS were evaluated for the safety of the study medication. Incidence, severity and causal relationship of any AEs were tabulated by system organ class (SOC) and preferred term (PT) coded according to MedDRA version 17.0. All AEs occurring between the time of the first drug intake and the drug discontinuation date as recorded in the eCRF plus 1 day (inclusive) were assigned to the respective treatment group. Adverse events occurring before the time of first drug intake were assigned to ‘screening’ and AEs occurring after the drug discontinuation date plus 1 day (inclusive) were assigned to ‘post‐treatment’. In addition, AEs with an onset date before the start of study drug but with worsening in intensity during treatment were assigned to the treatment period.

## 3 Results

### 3.1 Patients

A total of 748 patients were enrolled in this trial, of whom 562 patients were randomized and treated. The proportion of patients who discontinued trial medication prematurely was low (9 patients [1.6%]). The most common reason for discontinuation was other AE, which was reported in five patients (of whom 3 were treated with ibuprofen/caffeine–ibuprofen/caffeine). Two patients (0.4%) discontinued trial medication due to non‐compliance with the clinical trial protocol. The disposition of patients is summarized in Fig. 1.

Demographic characteristics (sex, race and ethnicity, age and BMI) and baseline pain intensities (VRS, NPRS) were well balanced across treatment arms (Supporting Information Table S1). Baseline pain intensity scores as measured on the VRS and the NPRS were balanced across treatment arms. More than half of patients (57.8%) reported ‘severe’ pain on the VRS; the remaining patients (42.2%) reported ‘moderate’ pain, and the mean pain score on the NPRS was 7.7. The baseline pain intensities were in the range of other trials using the dental impaction pain model (Forbes et al., 1991; Cheung et al., 2007).

Concomitant analgesic medications except for paracetamol (rescue medication, see below) were reported for <1% of the overall trial population and were balanced across treatment arms in Study Stage 1.

### 3.2 Efficacy

The adjusted mean SPRID0–8 h achieved with ibuprofen 400 mg/caffeine 100 mg was statistically significant higher when compared to all other treatment arms. The FDC provided approximately 30% higher pain reduction/pain relief than ibuprofen alone on average over 8 h. The SMDs for SPRID0–8 h were 1.4 and 1.2 for the comparisons FDC versus placebo and caffeine, respectively, and 0.4 for the comparison FDC versus ibuprofen alone.

The results of the primary analysis were confirmed by a sensitivity analysis using the per‐protocol set (data not shown) and corroborated by the results for the secondary endpoint SPRID0–2 h, which demonstrated about 50% higher pain reduction/pain relief with the FDC than with ibuprofen alone on average over the first 2 h after intake of the first dose of trial medication, i.e. in the immediate post‐surgical phase characterized by peaking acute pain (Table 2).

**Table 2:** Results for SPRID0–8 h and SPRID0–2 h – trial 1335.1, full analysis set (FAS)

	Placebo	Caffeine	Ibuprofen	Ibuprofen/caffeine
Number of patients, *N*	70	70	209	213
ANCOVA for SPRID_0–8 h_				
Adjusted mean (SE)	10.6 (3.5)	15.8 (3.5)	40.2 (2.0)	52.3 (2.0)
95% CI	(3.6, 17.5)	(8.9, 22.7)	(36.1, 44.2)	(48.3, 56.3)
Adjusted mean difference versus ibuprofen/caffeine (SE)	41.7 (4.1) 8.5 (0.8)	36.5 (4.1) 8.0 (0.8)	12.1 (2.9) 3.6 (0.6)	
95% CI	(33.8, 49.7)	(28.5, 44.4)	(6.5, 17.8)	
*p*‐Value	<0.0001	<0.0001	<0.0001	
ANCOVA for SPRID_0–2 h_				
Adjusted mean (SE)	2.1 (0.7)	2.6 (0.7)	7.0 (0.4)	10.6 (0.4)
95% CI	(0.7, 3.4)	(1.2, 4.0)	(6.2, 7.8)	(9.8, 11.4)
Adjusted mean difference versus ibuprofen/caffeine (SE)	8.5 (0.8)	8.0 (0.8)	3.6 (0.6)	
95% CI	(6.9, 10.1)	(6.4, 9.6)	(2.5, 4.7)	
*p*‐Value	<0.0001	<0.0001	<0.0001	

### 3.3 Pain intensity difference and pain relief at individual time points

**Figure 2:**
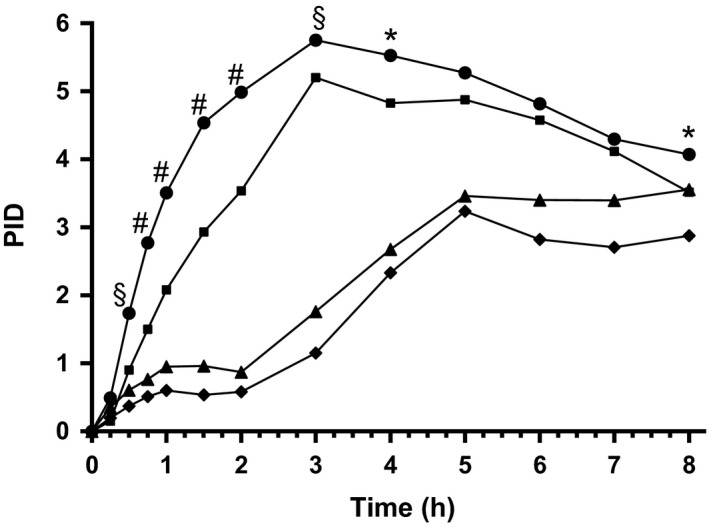
Adjusted means for pain intensity difference over time – full analysis set (FAS). Diamonds: placebo, triangles: caffeine, squares: ibuprofen, circles: ibuprofen/caffeine. Symbols on top of the ibuprofen/caffeine data indicate *p*‐values in comparison to ibuprofen at the given time points (^#^ *p* < 0.0001; ^§^ *p* < 0.001; *p < 0.05).

The analysis of pain intensity difference (as measured on the 0–10 NPRS) and pain relief (as measured on the 5‐point VRS) at individual time points corroborated the findings of the primary and secondary endpoint analyses. Treatment with ibuprofen/caffeine showed maintained analgesic efficacy with a fast onset. For pain intensity difference, the comparison of adjusted means for ibuprofen/caffeine versus ibuprofen achieved *p*‐values below 5% already after 0.5 h and up to 4 h of administration of trial medication. At 0.5 h the FDC reduced pain intensity by approximately 1.7 points compared to baseline whereas ibuprofen alone reduced PI by only 0.9 points. The time‐effect curves for pain intensity difference reflected a sustained treatment benefit over 8 h for ibuprofen/caffeine versus ibuprofen (Fig. 2). When comparing ibuprofen/caffeine with ibuprofen, the difference in pain intensity was greatest from 0.75 to 2 h (adjusted mean difference > 1.27) and peaked after 1.5 h (adjusted mean difference 1.6).

Concerning pain relief, the odds ratios for the comparison of ibuprofen/caffeine versus ibuprofen favoured the ibuprofen/caffeine arm already at the first time point after 0.25 h and up to 2 h of administration of trial medication (Supporting Information Table S2). The comparisons to placebo and caffeine are not shown here but are consistent with the results obtained for the primary and secondary endpoints described above.

### 3.4 Onset and duration of pain relief

**Figure 3:**
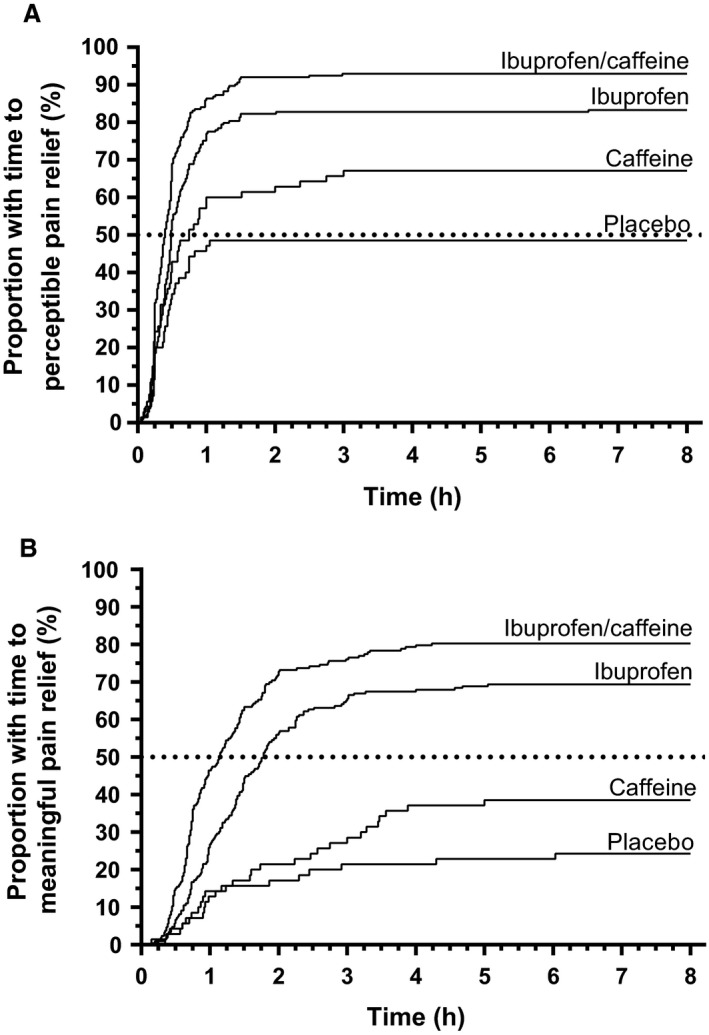
Kaplan–Meier estimates over time for time to onset of perceptible (A) and meaningful (B) pain relief.

The analyses for time to meaningful and time to perceptible pain relief showed consistent results. Median time to perceptible and median time to meaningful pain relief were shorter for treatment with ibuprofen/caffeine compared with ibuprofen, indicating that the onset of analgesic efficacy was faster with ibuprofen/caffeine than with ibuprofen (Fig. 3; Table 3). Most of patients in the placebo arm (75.7%) and caffeine arm (61.4%) did not achieve meaningful pain relief with their first dose of trial medication and were censored at 8 h.

**Table 3:** Kaplan–Meier analysis and log rank test for time to meaningful pain relief and time to perceptible pain relief – FAS

	Placebo	Caffeine	Ibuprofen	Ibuprofen/caffeine
Patients, *N*	70	70	209	213
Time to meaningful pain relief				
Median (95% CI) [h]	NC	NC	1.78 (1.48, 2.02)	1.13 (0.93, 1.35)
Comparison versus ibuprofen/caffeine *p*‐value^a^	<0.0001	<0.0001	0.0001	
Time to perceptible pain relief				
Median (95% CI) [h]	NC	0.75 (0.48, 1.52)	0.48 (0.47, 0.57)	0.40 (0.35, 0.45)
Comparison versus ibuprofen/caffeine *p*‐value^a^	<0.0001	<0.0001	0.0001	

NC = Not calculable (more than half of patients were without meaningful or perceptible pain relief within 8 h).

a Log rank test stratified for baseline pain intensity as measured on the 4‐point VRS.

The Kaplan–Meier curves for time to meaningful pain relief separated early, with the curves for ibuprofen and ibuprofen/caffeine separating after 0.25 h (in favour of ibuprofen/caffeine) and remaining separated over the entire observation period (see Fig. 3). The curves showed that the overall proportion of patients who reported meaningful pain relief was higher in the ibuprofen/caffeine arm than in the ibuprofen arm, suggesting that more patients responded to treatment with ibuprofen/caffeine than to ibuprofen. This observation was supported by the lower number of patients who were censored at 8 h (ibuprofen/caffeine: 19.7%, ibuprofen: 30.6%). The Kaplan–Meier curves for time to perceptible pain relief were similar to those for time to meaningful pain relief, with the proportions of patients who reported perceptible pain relief were higher than those for time to meaningful pain relief in the ibuprofen and ibuprofen/caffeine arms.

The median duration of pain relief (defined as the time until rescue medication or the second dose of trial medication was taken) was almost as long as the observation period of Study Stage 1 in the ibuprofen/caffeine arm (7.33 h, 95% CI: 7.05, 7.77 h), and ibuprofen arm (7.11 h, 95% CI: 6.43, 7.77 h), and significantly shorter in the caffeine (2.08 h, 95% CI: 1.67, 3.83 h) and placebo arms (1.63 h, 95% CI: 1.60, 2.07 h), respectively.

### 3.5 Use of rescue medication

The proportion of patients who took rescue medication in Study Stage 1 was more than twice as high in the ibuprofen arm (32.5%) compared with the ibuprofen/caffeine arm (16.0%) (Table 4).

**Table 4:** Distribution of patients by use of rescue medication in Study Stage 1 – FAS

	Placebo *N* (%)	Caffeine *N* (%)	Ibuprofen *N* (%)	Ibuprofen/caffeine *N* (%)	Total *N* (%)
Patients	70 (100.0)	70 (100.0)	209 (100.0)	213 (100.0)	562 (100.0)
Resue medication used					
None	17 (24.3)	25 (35.7)	141 (67.5)	179 (84.0)	362 (64.4)
1 dose	53 (75.7)	45 (64.3)	68 (32.5)	34 (16.0)	200 (35.6)

### 3.6 Responder analysis

An additional analysis was performed to derive responder rates from the results for SPRID0–8 h. The responder analysis for SPRID0–8 h confirmed the results of the primary endpoint analysis and the observation that more patients responded to treatment with ibuprofen/caffeine than to ibuprofen. Less than one quarter of patients in the placebo (17.3%) and caffeine (24.6%) arms achieved 50% of the maximum achievable SPRID0–8, whereas the 50% responder rates for ibuprofen and the FDC were 49.7% and 67.8%, respectively. Compared with placebo, treatment with ibuprofen/caffeine resulted in an ARR of 50.5% indicating an NNT of 2.0 (95% CI 1.6, 2.5; *p* < 0.0001) versus an NNT of 3.1 (95% CI 2.3, 4.7; *p* < 0.0001) for treatment with ibuprofen versus placebo.

Half of patients (49.7%) in the ibuprofen arm compared with two‐thirds of patients (67.8%) in the ibuprofen/caffeine arm achieved at least the half‐maximum achievable SPRID0–8 h with a number needed to treat (NNT) of 5.5, compared to ibuprofen. This means that the addition of caffeine to ibuprofen increased the proportion of patients who achieved at least the half‐maximal SPRID0–8 h by almost 20%, and that for every six patients treated with ibuprofen/caffeine, one would achieve 50% response who would not achieve it with ibuprofen. Similar results were obtained for the responder analysis of SPRID0–6 h (Table 5) and SPID0–6 h (Supporting Information Table S3).

**Table 5:** Responder analysis for SPRID0–6 h, FAS

	Ibuprofen/caffeine (*N* = 213)	Placebo (*N* = 70)	Caffeine (*N* = 70)	Ibuprofen (*N* = 209)
Number (%) with 50% response	150 (70.6)	11 (15.4)	15 (21.4)	110 (52.5)
95% CI [%]	(64.5, 76.7)	(7.0, 23.9)	(11.8, 31.0)	(45.7, 59.2)
Comparison versus Ibuprofen/caffeine				
ARR of Ibuprofen/caffeine versus comparator (95% CI)		55.2 (44.7, 65.6)	49.2 (37.8, 60.6)	18.1 (9.0, 27.3)
NNT of Ibuprofen/caffeine versus comparator (95% CI)		1.8 (1.5, 2.2)	2.0 (1.7, 2.6)	5.5 (3.7, 11.1)
*p*‐Value		<0.0001	<0.0001	<0.0001

### 3.7 Adverse events

Exposure of patients to placebo and caffeine was limited to a single tablet during Study Stage 1 which lasted 6–8 h. Therefore, AE frequencies are provided separately for Study Stage 1 and Stage 2.

The frequency of patients with any AE in Study Stage 1 was 6.1% for ibuprofen/caffeine, 2.4% for ibuprofen, 2.9% for caffeine and 4.3% for placebo. Most patients experienced mild AEs, none had a serious AE. In the ibuprofen/caffeine group 3 subjects had a total of 3 AEs (nausea, *n* = 2 and headache, *n* = 1) which were considered drug‐related versus 1 subject with nausea in the ibuprofen group, none in the placebo group and 2 subjects with a total of 2 AEs (nausea, *n* = 1 and vomiting, *n* = 1) in the caffeine group. One patient in the ibuprofen group experienced an AE of severe intensity (vomiting). None of the AEs led to treatment discontinuation.

The most frequently (>1% in at least one treatment group) reported AEs on preferred term (PT) level in Study Stage 1 were nausea (placebo: 4.3%, caffeine: 1.4%, ibuprofen: 1.4%, ibuprofen/caffeine: 3.8%) and vomiting (placebo: 0%, caffeine: 1.4%, ibuprofen: 0.5%, ibuprofen/caffeine: 0%).

In Study Stage 2, the patients allocated to placebo and caffeine received either ibuprofen/caffeine or ibuprofen 3 tablets daily up to 5 days. The frequency of patients with any AE in Study Stage 2 was 21.6% for ibuprofen/caffeine and 13.3% for ibuprofen. The majority of patients were reported with AEs of mild or moderate severity, severe AEs occurred in 2 patients (0.7%) in both treatment arms. AEs considered as drug‐related by the investigator were experienced by 11 patients of the ibuprofen/caffeine arm (3.9%) and two patients of the ibuprofen arm (0.7%). In one patient of the ibuprofen arm (0.4%) and four patients of the ibuprofen/caffeine arm (1.4%) AEs resulted in the discontinuation of study drug. One patient receiving ibuprofen discontinued due to a serious adverse event (SAE) requiring hospitalization. The SAE (oral infection of severe intensity) was considered to be not related to study drug intake; the patient recovered. None of the drug‐related AEs were considered to be serious and all patients recovered from all drug related AEs, except for one patient who was lost to follow‐up. All AEs leading to discontinuation that were reported in the ibuprofen/caffeine arm were non‐serious and of mild or moderate intensity.

The most frequently reported (>1% in at least one treatment group) AEs on preferred term (PT) level in Study Stage 2 were nausea (ibuprofen: 2.5%, ibuprofen/caffeine: 5.3%), aphthous stomatitis (ibuprofen: 0.7%, ibuprofen/caffeine: 1.4%), vomiting (ibuprofen: 1.1%, ibuprofen/caffeine: 1.1%), alveolar osteitis (ibuprofen: 1.8%, ibuprofen/caffeine: 2.8%), dizziness (ibuprofen: 0.7%, ibuprofen/caffeine: 3.5%), headache (ibuprofen: 0.4%, ibuprofen/caffeine: 1.4%) and insomnia (ibuprofen: 0.7%, ibuprofen/caffeine: 4.6%).

### 3.8 Patient assessment of tolerability

At the end of Study Stage 2, patients rated the tolerability of the study medication as ‘very good’ (ibuprofen: 36.9%, ibuprofen/caffeine: 38.7%) or ‘excellent’ (ibuprofen: 33.3%, ibuprofen/caffeine: 30.9%). A comparison of ibuprofen versus ibuprofen/caffeine resulted in an odds ratio of 1.2, slightly in favour of the FDC.

## 4 Discussion

This phase III, randomized, active‐ and placebo‐controlled, double‐blind study demonstrated superior efficacy of the FDC 400 mg ibuprofen and 100 mg caffeine over either single compound and placebo in the dental impaction model, which is validated, reproducible and predictable for treatments of acute pain in general. The results confirm recent meta‐analyses which have shown that 100–130 mg caffeine used as analgesic adjuvant for decades increases the responder rate of WHO stage I analgesics (Derry et al., 2014, 2015).

With regard to the primary efficacy endpoint the FDC ibuprofen/caffeine provided approximately 30% higher pain reduction/pain relief than ibuprofen alone on average over 8 h. The achieved SMDs for SPRID0–8 h were 1.4 and 1.2 for the comparisons FDC versus placebo and caffeine, respectively, and 0.4 for the comparison FDC versus ibuprofen alone, i.e. they exceeded the minimum clinically relevant effects sizes used for the sample size calculation, so that the results of the chosen primary endpoint are clinically relevant and statistically significant. PID over time showed that up to 4 h after intake, as well as at the time point 8 h there was a relevant supplemental effect of caffeine in combination with ibuprofen when compared to ibuprofen alone. Clinical trials investigating various doses of ibuprofen for the treatment of acute pain showed that at 400 mg single dose a ceiling effect is reached, i.e. a higher ibuprofen dose does not induce more pronounced pain reduction, although higher peak plasma levels can be achieved (Laska et al., 1986; Seymour et al., 1996; Kellstein et al., 2000).

Within the 8 h observation period in Study Stage 1, duration of pain relief (as assessed by the time until intake of rescue medication or the second dose of test medication) was slightly longer for the FDC compared to ibuprofen. It can be speculated that this might have been induced by the relative fast recovery of the patients (as seen in the placebo and caffeine arm), which in this trial was faster compared to other published data (see e.g. Seymour et al., 1996; Mehlisch et al., 2010).

The analysis of various endpoints showed that the FDC provided faster pain reduction than ibuprofen alone: Pain relief was significantly different from ibuprofen as early as 15 min after intake (Supporting Information Table S2), and the same was the case for PID after 30 min (Fig. 2). Median time to meaningful pain relief was 0.65 h (39 min) earlier for patients taking the FDC, compared to those taking ibuprofen (Fig. 3, Table 3). Moore and colleagues performed a meta‐analysis comparing pharmacokinetic and pharmacodynamic differences between standard oral formulations of ibuprofen and fast release forms (like ibuprofen formulated as arginate, lysinate or sodium salt; [Moore et al., 2014]): the investigated studies reported differences of 4 min (400 mg ibuprofen as sodium salt vs. ibuprofen acid; [Norholt et al., 2011]) to 27 min (400 mg ibuprofen as arginate vs. ibuprofen acid; [Mehlisch et al., 2002]). Comparing to these literature data on very fast releasing salts, meaningful pain relief was even achieved faster by the FDC ibuprofen/caffeine in this study compared to ibuprofen acid.

In accordance with the study protocol, PI and PAR data were set missing after a patient had taken rescue/second study medication, and the LOCF procedure was used for summary endpoints (SPRID, SPID, TOTPAR), following previously published clinical studies using such endpoints and their analyses. In acute pain, baseline observation carried forward was described to be more conservative than last observation carried forward, which was considered relevant for trials longer than 8 h (Moore et al., 2005). However, in this study with data cut‐off at 8 and 6 h, sensitivity analysis using the observed PI data, regardless of additional analgesics taken, as well as an analysis using placebo‐multiple‐imputation for data at time points following intake of additional analgesics revealed statistically significant superiority of the FDC versus the other treatment groups. Moreover, it should be taken into account that the remedication rate was lowest for the FDC, and therefore baseline observation carried forward would have been less conservative than LOCF.

In their meta‐analysis on standard and very fast release forms of ibuprofen, Moore and colleagues showed that the latter provided more benefits to the patients reflected by higher responder rates (defined as percentage of patients with ≥50% total pain relief over 0–6 h) and a lower proportion of patients who took an additional dose of analgesic within the observation period (Moore et al., 2014). In their analysis, for standard release ibuprofen (400 mg), 53% of patients in the dental pain model experienced ≥50% total pain relief over 0–6 h, compared to 66% for fast release formulations. In our study, the figures were 52.5% for standard ibuprofen, and 70.6% for the FDC (Table 5). Thus, the outcomes for standard ibuprofen were comparable to published data while for the FDC these outcomes were even more favourable than those published for very fast release formulations.

Results well comparable to published data were also obtained for the remedication rate: Moore and colleagues reported that 43% of patients on standard ibuprofen versus 32% of patients taking fast release ibuprofen remedicated within 6 h in the dental model. According to the results obtained in our study 32.5% of patients in the ibuprofen arm remedicated compared to 16.0% in the FDC arm. The data obtained in this study are consistent as well with those reported for other pain models in the literature, e.g. for the treatment of tension type headache with 400 mg ibuprofen/200 mg caffeine (in comparison to ibuprofen, caffeine and placebo; [Diamond et al., 2000]) showed that the adjuvant effect of caffeine is not restricted to ibuprofen's analgesic properties on dental extraction pain.

The FDC also compares well to other medicinal products that have been investigated in the dental extraction model: total pain relief over 0–6 h was achieved in 70.6% of patients after FDC. Only high doses of etoricoxib (≥120 mg) and ketoprofen 100 mg provided total pain relief over 0–6 h in 71–79% of patients (etoricoxib) and >72% (ketoprofen) (Moore et al., 2011).

Overall, the number of patients with adverse events was low in this study, and the FDC ibuprofen/caffeine was shown to be well tolerated and safe even over a period of 5 days. The majority of these events were of mild or moderate intensity with the most frequently reported AEs being nausea and vomiting. The most frequent (drug‐related) AEs nausea, vomiting, insomnia and dizziness were expected and are known side effects of ibuprofen or caffeine. Also insomnia is a common side effect of caffeine intake and alveolar osteitis is a common sequel after third molar extraction. The patients rating of overall tolerability was similar under treatment with ibuprofen and ibuprofen/caffeine slightly favouring the FDC, however, in both groups about 70% of the patients rated the tolerability as ‘very good’ or ‘excellent’.

In summary, this study demonstrated that the FDC 400 mg ibuprofen/100 mg caffeine is an effective treatment for acute pain and is well tolerated and safe when administered over a period of five consecutive days, with superior efficacy compared to 400 mg ibuprofen alone.

## Author contributions

T.W., E.R., A.H., D.D.M. and R.L. substantially contributed to study concept, design and results interpretation. The manuscript was drafted by T.W., E.R., A.H. and R.L. All authors critically revised the manuscript for important intellectual content and approved it.

## Study registration number

ClinicalTrials.gov Identifier: NCT01929031.

## Supporting information


**Table S1.** Demographics and baseline pain intensities in Study Stage 1 – treated set (TS).

**Table S2.** Pain relief at individual time points compared with ibuprofen/caffeine– FAS.

**Table S3.** Responder analysis for SPID0–6 h, FAS.

## References

[ejp1068-bib-0001] Cheung, R. , Krishnaswami, S. , Kowalski, K. (2007). Analgesic efficacy of celecoxib in postoperative oral surgery pain: A single‐dose, two‐center, randomized, double‐blind, active‐ and placebo‐controlled study. Clin Ther 29, 2498–2510. 18164917 10.1016/j.clinthera.2007.12.008

[ejp1068-bib-0002] Cooper, S.A. , Desjardins, P.J. (2010). The value of the dental impaction pain model in drug development. Methods Mol Biol 617, 175–190. 20336423 10.1007/978-1-60327-323-7_15

[ejp1068-bib-0003] Derry, C.J. , Derry, S. , Moore, R.A. (2014). Caffeine as an analgesic adjuvant for acute pain in adults (review). Cochrane Database Syst Rev, CD009281. 25502052 10.1002/14651858.CD009281.pub3 PMC6485702

[ejp1068-bib-0004] Derry, S. , Wiffen, P.J. , Moore, R.A. (2015). Single dose oral ibuprofen plus caffeine for acute postoperative pain in adults (review). Cochrane Database Syst Rev, CD011509. 26171993 10.1002/14651858.CD011509.pub2 PMC6481458

[ejp1068-bib-0005] Diamond, S. , Balm, T.K. , Freitag, F.G. (2000). Ibuprofen plus caffeine in the treatment of tension‐type headache. Clin Pharmacol Ther 68, 312–319. 11014413 10.1067/mcp.2000.109353

[ejp1068-bib-0006] Forbes, J.A. , Beaver, W.T. , Jones, K.F. , Kehm, C.J. , Gongloff, C.M. , Zeleznock, J.R. , Smith, J.W. (1991). Effect of caffeine on ibuprofen analgesia in postoperative oral surgery pain. Clin Pharmacol Ther 49, 674–684. 2060256 10.1038/clpt.1991.85

[ejp1068-bib-0007] Kellstein, D.E. , Lipton, R.B. , Geetha, R. , Koronkiewicz, K. , Evans, F.T. et al. (2000). Evaluation of a novel solubilized formulation of ibuprofen in the treatment of migraine headache: A randomized, double‐blind, placebo‐controlled, dose‐ranging study. Cephalalgia 20, 233–243. 10999673 10.1046/j.1468-2982.2000.00055.x

[ejp1068-bib-0008] Laska, E.M. , Sunshine, A. , Marrero, I. , Olson, N. , Siegel, C. , McCormick, N. (1986). The correlation between blood levels of ibuprofen and clinical analgesic response. Clin Pharmacol Ther 40, 1–7. 3522030 10.1038/clpt.1986.129

[ejp1068-bib-0009] Mehlisch, D.R. , Ardia, A. , Pallotta, T. (2002). A controlled comparative study of ibuprofen arginate versus conventional ibuprofen in the treatment of postoperative dental pain. J Clin Pharmacol 42, 904–911. 12162473 10.1177/009127002401102821

[ejp1068-bib-0010] Mehlisch, D.R. , Aspley, S. , Daniels, S.E. , Southerden, K.A. , Christensen, K.S. (2010). A single‐tablet fixed‐dose combination of racemic ibuprofen/paracetamol in the management of moderate to severe postoperative dental pain in adult and adolescent patients: A multicenter, two‐stage, randomized, double‐blind, parallel‐group, placebo‐controlled, factorial study. Clin Ther 32, 1033–1049. 20637958 10.1016/j.clinthera.2010.06.002

[ejp1068-bib-0011] Moore, R.A. , Edwards, J.E. , McQuay, H.J. (2005). Acute pain: Individual patient meta‐analysis shows the impact of different ways of analysing and presenting results. Pain 116, 322–331. 15979792 10.1016/j.pain.2005.05.001

[ejp1068-bib-0012] Moore, R.A. , Derry, S. , McQuay, H.J. , Wiffen, P.J. (2011). Single dose oral analgesics for acute postoperative pain in adults (review). Cochrane Database Syst Rev, CD008659. 21901726 10.1002/14651858.CD008659.pub2 PMC4160790

[ejp1068-bib-0013] Moore, R.A. , Derry, S. , Straube, S. , Ireson‐Paine, J. , Wiffen, P.J. (2014). Faster, higher, stronger? Evidence for formulation and efficacy for ibuprofen in acute pain. Pain 155, 14–21. 23969325 10.1016/j.pain.2013.08.013

[ejp1068-bib-0014] Norholt, S.E. , Hallmer, F. , Hartlev, J. , Pallesen, L. , Blomlof, J. et al. (2011). Analgesic efficacy with rapidly absorbed ibuprofen sodium dihydrate in postsurgical dental pain: Results from the randomized QUIKK trial. Int J Clin Pharmacol Ther 49, 722–729. 22122814 10.5414/cp201553

[ejp1068-bib-0015] Rainsford, K.D. (2009). Ibuprofen: Pharmacology, efficacy and safety. Inflammopharmacology 17, 275–342. 19949916 10.1007/s10787-009-0016-x

[ejp1068-bib-0016] Sawynok, J. (2011). Methylxanthines and pain. Handb Exp Pharmacol 200, 311–329. 10.1007/978-3-642-13443-2_11 20859801

[ejp1068-bib-0017] Seymour, R.A. , Ward‐Booth, P. , Kelly, P.J. (1996). Evaluation of different doses of soluble ibuprofen and ibuprofen tablets in postoperative dental pain. Br J Oral Maxillofac Surg 34, 110–114. 8645662 10.1016/s0266-4356(96)90147-3

[ejp1068-bib-0018] Singla, N.K. , Desjardins, P.D. , Chang, P.D. (2014). A comparison of the clinical and experimental characteristics of four acute surgical pain models: Dental extraction, bunionectomy, joint replacement, and soft tissue surgery. Pain 155, 441–456. 24012952 10.1016/j.pain.2013.09.002

