# Supplementary material for: Efficacy and safety of a fixed‐dose combination of ibuprofen and caffeine in the management of moderate to severe dental pain after third molar extraction
Source: Eur J Pain. 2017 Aug 14;22(1):28–38. doi: 10.1002/ejp.1068 (PMC5763370; doi:10.1002/ejp.1068)
Supplement: Supplementary file 1 — Table S1. Demographics and baseline pain intensities in Study Stage 1 – treated set (TS). [file EJP-22-28-s001.docx]

Supplementary Table 1

Demographics and baseline pain intensities in Study Stage 1 – treated set (TS)

|  | Placebo | Caffeine | Ibuprofen | Ibuprofen/ caffeine | Total |
| --- | --- | --- | --- | --- | --- |
| Number of patients [N (%)] | 70 (100.0) | 70 (100.0) | 209 (100.0) | 213 (100.0) | 562 (100.0) |
| Sex [N (%)]  Male  Female | 24 (34.3)  46 (65.7) | 31 (44.3)  39 (55.7) | 67 (32.1)  142 (67.9) | 82 (38.5)  131 (61.5) | 204 (36.3)  358 (63.7) |
| Race [N (%)]  White  Other | 68 (97.1)  2 (2.9) | 68 (97.1)  2 (2.9) | 198 (94.7)  11 (5.3) | 201 (94.4)  12 (5.6) | 535 (95.2)  27 (4.8) |
| Ethnicity [N (%)]  Not Hispanic/Latino  Hispanic/Latino | 63 (90.0)  7 (10.0) | 66 (94.3)  4 (5.7) | 183 (87.6)  26 (12.4) | 190 (89.2)  23 (10.8) | 502 (89.3)  60 (10.7) |
| Age [years]  Mean (SD) | 19.3 (1.77) | 19.2 (1.72) | 19.6 (1.96) | 19.5 (2.06) | 19.5 (1.95) |
| VRS baseline pain intensity [N (%)]  Moderate  Severe | 29 (41.4)  41 (58.6) | 30 (42.9)  40 (57.1) | 88 (42.1)  121 (57.9) | 90 (42.3)  123 (57.7) | 237 (42.2)  325 (57.8) |
| NPRS baseline pain intensity [N (%)]  Mean (SD)  Median  Range (min, max) | 7.8 (0.89)  8.0  (5, 10) | 7.5 (1.11)  7.0  (5, 10) | 7.8 (1.10)  8.0  (5, 10) | 7.8 (1.13)  8.0  (5, 10) | 7.7 (1.09)  8.0  (5, 10) |
| BMI [kg/m^2^]  Mean (SD) | 23.81 (2.869) | 22.84 (3.058) | 23.36 (3.101) | 23.14 (3.004) | 23.27 (3.034) |
