# Supplementary material for: Efficacy and safety of a fixed‐dose combination of ibuprofen and caffeine in the management of moderate to severe dental pain after third molar extraction
Source: Eur J Pain. 2017 Aug 14;22(1):28–38. doi: 10.1002/ejp.1068 (PMC5763370; doi:10.1002/ejp.1068)
Supplement: Supplementary file 2 — Table S2. Pain relief at individual time points compared with ibuprofen/caffeine– FAS. [file EJP-22-28-s002.docx]

Supplementary Table 2

Pain relief at individual time points compared with ibuprofen/caffeine– FAS

|  | Ibuprofen (N = 209) | |
| --- | --- | --- |
| Time point | Odds ratio (95% CI) | p-value |
| 0.25 h | 2.0 (1.3, 3.0) | 0.0016 |
| 0.5 h | 1.9 (1.4, 2.7) | <0.0001 |
| 0.75 h | 2.5 (1.8, 3.5) | <0.0001 |
| 1 h | 2.9 (2.0, 4.1) | <0.0001 |
| 1.5 h | 2.9 (2.0, 4.3) | <0.0001 |
| 2 h | 2.9 (2.0, 4.3) | <0.0001 |
| 3 h | 1.4 (1.0, 2.1) | 0.0634 |
| 4 h | 1.3 (0.9, 2.0) | 0.1335 |
| 5 h | 1.1 (0.7, 1.6) | 0.7129 |
| 6 h | 0.9 (0.6, 1.4) | 0.7135 |
| 7 h | 0.9 (0.6, 1.3) | 0.4472 |
| 8 h | 1.1 (0.7, 1.7) | 0.7532 |
