# Supplementary material for: Efficacy and safety of a fixed‐dose combination of ibuprofen and caffeine in the management of moderate to severe dental pain after third molar extraction
Source: Eur J Pain. 2017 Aug 14;22(1):28–38. doi: 10.1002/ejp.1068 (PMC5763370; doi:10.1002/ejp.1068)
Supplement: Supplementary file 3 — Table S3. Responder analysis for SPID0–6 h, FAS. [file EJP-22-28-s003.docx]

Supplementary Table 3

Responder analysis for SPID0-6h, FAS

|  | Ibuprofen/ caffeine (N=213) | Placebo (N=70) | Caffeine (N=70) | Ibuprofen (N=209) |
| --- | --- | --- | --- | --- |
| Number (%) with 50% response  95% confidence interval [%] | 150 (70.6)  (64.5, 76.7) | 10 (15.0)  (6.6, 23.3) | 14 (20.5)  (11.1, 30.0) | 105 (50.2)  (43.5, 57.0) |
| **Comparison versus Ibuprofen/caffeine**  ARR of Ibuprofen/caffeine vs comparator  95% confidence interval  **NNT of Ibuprofen/caffeine vs comparator** 95% confidence interval  p-value |  | 55.7  (45.3, 66.0)  **1.8**  (1.5, 2.2)  <0.0001 | 50.1  (38.8, 61.4)  **2.0**  (1.6, 2.6)  <0.0001 | 20.4  (11.3, 29.5)  **4.9**  (3.4, 8.9)  <0.0001 |
